# Immunisierung unter Knappheitsbedingungen – Norm und Praxis des Impfens in der DDR 1949 bis 1970

**DOI:** 10.1007/s00103-025-04028-2

**Published:** 2025-03-06

**Authors:** Anne Thordis Wanke, Florian Bruns

**Affiliations:** Institut für Geschichte der Medizin, Medizinische Fakultät Carl Gustav Carus, Technische Universität Dresden, Fetscherstraße 74, 01307 Dresden, Deutschland

**Keywords:** Impfung, Poliomyelitis, DDR, Medizingeschichte, Ressourcenallokation, Vaccination, Poliomyelitis, German Democratic Republic, History of medicine, Resource allocation

## Abstract

In der Deutschen Demokratischen Republik (DDR) etablierte sich in den 1950er- und 1960er-Jahren ein straff organisiertes Impfwesen. Dabei paarte sich das medizinische Bemühen, die in der Nachkriegszeit verbreiteten Infektionskrankheiten zu stoppen, mit dem politischen Willen, sich als verantwortungsvoller Vorsorgestaat zu präsentieren. Anhand von unpubliziertem Archivmaterial beleuchten wir im Folgenden die Alltagspraxis des Impfens in der frühen DDR. Wir können zeigen, dass die Vorgaben beim Impfen durch Materialengpässe, Personal- und Devisenmangel nur schwer zu erreichen waren. Häufig mussten die politisch gesetzten Normen an die Alltagsgegebenheiten angepasst und pragmatische Lösungen gefunden werden. Zu den Strategien im Umgang mit knappen Impfressourcen gehörten u. a. die Delegierung des Impfens an nichtärztliches Personal sowie die impfstoffsparende intrakutane Applikation. Impfmüdigkeit in der Bevölkerung war auch in der DDR ein Thema, das die Gesundheitsbehörden beschäftigte. Zur Erzielung hoher Impfquoten führte das DDR-Gesundheitsministerium Leistungsvergleiche auf Kreis- und Bezirksebene ein. Zudem wurde die Aufnahme in Kindergärten und Ferienlager an entsprechende Impfnachweise gebunden. Weitergehende Sanktionen, wie etwa Geldstrafen, wurden nur selten verhängt.

## Abstract (en)

In the German Democratic Republic (GDR), a tightly organised vaccination system was established in the 1950s and 1960s. In this context, the medical effort to stop the spread of infectious diseases in the post-war period was coupled with the political will to present itself as a responsible preventive state. Based on unpublished archival material, we will shed light on the everyday practice of vaccination in the early GDR. We can show that the vaccination targets were difficult to achieve due to material shortages, personnel shortages and a lack of foreign currency. Often, the politically set norms had to be adapted to everyday realities and pragmatic solutions had to be found. Strategies for dealing with scarce vaccination resources included delegating vaccinations to non-medical personnel and using intradermal application to save vaccine. Vaccination fatigue in the population was also an issue for the health authorities in the GDR. To achieve high vaccination rates, the GDR Ministry of Health introduced performance comparisons at the district and county level. In addition, admission to daycare centres and holiday camps was made contingent upon the presentation of the relevant vaccination certificates. Further sanctions, such as fines, were rarely imposed.

## Einleitung

Das vor allem an prophylaktischen Leitideen orientierte staatliche Gesundheitswesen der Deutschen Demokratischen Republik (DDR) galt als eine Vorzeigeerrungenschaft des real existierenden Sozialismus. Aus medizinisch-epidemiologischen wie aus politisch-ideologischen Gründen etablierte die DDR ein straff organisiertes Impfwesen mit (vermeintlich) umfassendem Zugriff auf die Bevölkerung. Dabei paarte sich das Bemühen, die in der Nachkriegszeit verbreiteten Infektionskrankheiten zu stoppen, mit dem Willen der Sozialistischen Einheitspartei Deutschlands (SED), die DDR nach innen wie nach außen als verantwortungsvollen, gut organisierten Vorsorgestaat zu präsentieren. Hohe Impfquoten galten als Beleg für die Funktionalität und Bindungskraft des Sozialismus [1]. Erfolge bei der Zurückdrängung übertragbarer Krankheiten trugen zur Legitimation des sozialistischen Systems bei und wirken bis heute im kollektiven Gedächtnis nach. Dass der „sozialistische Gesundheitsschutz“ (so der offiziell verwandte Begriff) auch eine Impfpflicht für verschiedene Schutzimpfungen einschloss, hat der zumeist positiven Erinnerung an das Impfen in der DDR keinen Abbruch getan, mag das Misstrauen gegenüber heutigen Impfkampagnen auch noch so groß sein.

Umso wichtiger erscheint ein historisch differenzierter Blick auf das ostdeutsche Impfregime, das bisher nur ansatzweise erforscht ist. Insbesondere über den Alltag des Impfens und den Umgang mit der auch in der DDR vorhandenen Impfskepsis ist noch wenig bekannt [2]. Unser Beitrag beabsichtigt, die Impfpraxis in der DDR etwas genauer zu beleuchten und sie anhand von neu erschlossenem Archivmaterial und gestützt auf die spärliche Forschungsliteratur mit den staatlichen Vorgaben zu vergleichen. Wir konzentrieren uns dabei auf die 1950er- und 1960er-Jahre, in denen sich das Impfwesen der DDR im Aufbau befand. Basierend auf unserer regional angelegten Studie zur Impfkampagne gegen Poliomyelitis in Halle (Saale) [3] können wir nun aus größerem Blickwinkel zeigen, dass politisch geforderte Zielvorgaben beim Impfen durch Devisenmangel, Materialengpässe und Personalknappheit erheblich erschwert wurden. Um die daraus resultierenden Konflikte zu lösen, mussten die politisch gesetzten Normen häufig an die Alltagsgegebenheiten angepasst und pragmatische Lösungen gefunden werden.

## Politik und Bürokratie des Impfens in der DDR

Nach Gründung der DDR 1949 wurde der bereits in den Vorjahren begonnene Aufbau eines staatlichen, zentral gelenkten Gesundheitswesens fortgesetzt. Seine theoretische Grundlage speiste sich aus verschiedenen Einflüssen: Vorgaben der Sowjetischen Militäradministration in Deutschland (SMAD) mischten sich mit sozialhygienischen Konzepten der Weimarer Republik [4, 5]. Die alarmierende gesundheitliche Lage der Bevölkerung in den unmittelbaren Nachkriegsjahren erzwang überdies eine effiziente Allokation der knappen personellen und materiellen Ressourcen. Der sich aus dieser epidemiologischen Gründungskrise entwickelnde gesundheitspolitische Ansatz erstrebte die „Einheit von Prophylaxe, Diagnostik, Therapie und Metaphylaxe“, um die Gesundheit der Bevölkerung zu erhalten und zu heben [6]. Insbesondere Ärztinnen und Ärzte sollten „Gesundheitsinstrukteure und -kontrolleure sein, nicht nur Reparaturbrigade“ [7]. Unter der in den 1950er-Jahren eingeführten Losung „Der Sozialismus ist die beste Prophylaxe“ (variiert als: „Die beste Prophylaxe ist der Sozialismus“), festigte sich ein Primat präventiver Medizin mit Impfprogrammen als tragender Säule [1]. Bereits 1946 war das Impfen als zentrale Aufgabe beim „Aufbau des neuen Deutschlands“ definiert worden [8]. Prävention mittels Impfens versprach, medizinische Ressourcen zu schonen und teure Behandlungen zu vermeiden. Angesichts vergleichsweise moderater Beschaffungskosten konnten Impfungen Kindern, Jugendlichen und Erwachsenen aller Schichten angeboten werden, was dem sozialistischen Gleichheitsgedanken bestens entsprach. Auch das epidemiologische Ziel der Herdenimmunität mutet aus übergeordneter Perspektive wie ein Gleichnis auf die sozialistische Gesellschaftsbetrachtung an, in der das Wohl des Kollektivs zumeist Priorität gegenüber individuellen Gesundheitsentscheidungen besaß.

Schaltzentrale der Gesundheits- und damit Impfpolitik war das 1950 gegründete Ministerium für Gesundheitswesen (MfG), das wiederum der Abteilung Gesundheitspolitik der SED unterstellt und berichtspflichtig war. Erarbeitet wurden die zentralen Impfprogramme und Planvorgaben von der Staatlichen Hygiene-Inspektion (SHI), einer Hauptabteilung des MfG [9]. Die mittlere staatliche Verwaltungsebene bestand seit 1952 aus Bezirken, die von Räten geleitet wurden. Den dort angesiedelten Abteilungen für Gesundheits- und Sozialwesen standen jeweils eine Bezirksärztin oder ein Bezirksarzt vor. Ein ähnlicher Aufbau bestand auf der Ebene der Kreise. Diese untere Verwaltungsebene war die entscheidende Instanz für die lokale Umsetzung der Impfvorgaben; hier wurden Impfpersonal, Räume, Utensilien und Termine organisiert und koordiniert [10].

## Aufbau des Impfwesens

Im Zuge der Wochenbettnachsorge erhielten Eltern eine Woche nach Geburt eines Kindes Besuch von staatlichen Gesundheitsfürsorgerinnen, die auch den Impfausweis des Kindes überbrachten, ein wichtiges Dokument im Leben von DDR-Bürgern, das in vielen Familien heute noch überliefert ist. Innerhalb der ersten vier Lebenswochen hatte zudem eine erste Vorstellung von Säugling und Wöchnerin in einer „Beratungsstelle für Mutter und Kind“ zu erfolgen. Diese Beratungsstellen waren Teil des umfangreichen, in den 1950er-Jahren etablierten Fürsorgenetzes und berieten sowohl zu reproduktiver als auch zu kindlicher Gesundheit. Angebunden war in der Regel eine sogenannte Dauerimpfstelle. Diese ab 1953 zur Durch- bzw. Weiterführung der Pockenschutzimpfung aufgebauten Einrichtungen gingen aus den staatlichen Impfstellen hervor, die sich seit Einführung der Pflichtimpfung gegen Pocken im Jahr 1874 im Deutschen Reich etabliert hatten [1]. Die Impfstellen hatten sich als derart förderlich für die Impfbeteiligung erwiesen, dass ihre Zahl ab 1954 weiter erhöht wurde, um niedrigschwellig neben einer allgemeinen Impfberatung auch weitere Schutzimpfungen anbieten zu können. Häufig besaßen auch Polikliniken oder Ambulatorien eine Dauerimpfstelle. Die individuelle Beratung und Immunisierung in Dauerimpfstellen sollten perspektivisch die Praxis früherer Massenimpfungen in Schulen oder Gasthäusern ablösen, die unter hygienischen Aspekten als nicht mehr vertretbar galten. Zumindest Schulen und Betriebe blieben jedoch bis in die 1980er-Jahre hinein bevorzugte Orte der Immunisierung [1].

Ab dem dritten Lebensjahr gingen gesundheitliche Betreuung – und damit die Durchführung weiterer Impfungen – von den Mütterberatungsstellen an die Kreisabteilungen für Kinder- und Jugendgesundheitsschutz über. Diese blieben zuständig, bis ein Kind den Schulabschluss erreicht hatte. In den Bildungs- und Betreuungseinrichtungen angestelltes medizinisches Personal führte wiederum regelmäßig Reihenuntersuchungen und Impfungen durch, ohne dass Gesundheitseinrichtungen eigens aufgesucht werden mussten [10]. Die regelhafte Verknüpfung medizinischer Strukturen mit dem Betreuungs- und Bildungswesen erleichterte wesentlich den Zugriff auf Kinder und Jugendliche als potenzielle Impflinge. Gleiches galt für das Betriebsgesundheitswesen, über das die Masse der (erwachsenen) Arbeitnehmer erreicht wurde.

Zentral dokumentiert wurden die Impfungen auf „Karteikarten über Schutzimpfungen (außer BCG)“^1^. Geführt von kreisbeauftragten Impfsachbearbeiterinnen, lagen sie den Dauerimpfstellen vor, die qua Impfbezirk für den Wohnort des Impflings zuständig waren [11]. Im Rahmen der statistischen Impfauswertung des MfG ließen sich aus den Daten nicht nur epidemiologische Entwicklungen entnehmen, sondern auch die Planerfüllung im Bereich des Impfwesens überprüfen. Es resultierte ein kreis- und bezirksübergreifender Leistungsvergleich, der gleichsam in eine Form des Wettbewerbs mündete. Ab 1967 wurden die Ergebnisse des Leistungsvergleichs in Ranglistenform veröffentlicht und als Maß sowohl für die Arbeit der lokalen Gesundheitsfunktionäre als auch für die Zustimmung der Bevölkerung zum Impfen gewertet [1].

## Pflichten und Rechte der Impflinge

Die Verknüpfung von Impfprävention und sozialistischer Politik machte Impfquoten zu einem Gradmesser für die Loyalität der Bevölkerung zum SED-Staat. Nach dieser Logik musste die Staats- und Parteiführung das Nichterreichen vorab festgelegter Impfquoten als eine Art politischen Misstrauensbeweis deuten [1]. Angesichts einer allmählich einsetzenden Impfmüdigkeit der Bevölkerung, begünstigt durch den Rückgang zahlreicher Infektionskrankheiten, gerieten staatliche Impfprogramme ab Mitte der 1960er-Jahre nochmals stärker unter Erfolgsdruck. Der Ausweg wurde in erweiterten staatlichen Interventionsrechten gesucht: Zunehmend wurden Impfungen nicht mehr auf freiwilliger, sondern auf verpflichtender Basis geplant und durchgeführt.

Bereits in der sowjetischen Besatzungszone hatte es in den damals noch bestehenden Ländern regionale Pflichtimpfungen gegen Infektionskrankheiten gegeben. So war beispielsweise in Thüringen ab August 1946 die Ausgabe von Lebensmittelkarten an einen Immunisierungsnachweis gegen Typhus gebunden [12]. Die erste nationale Pflichtimpfung der DDR entstand durch Übernahme des Reichsimpfgesetzes von 1874, das die Impfpflicht gegen Pocken festschrieb, an der die DDR bis 1982 festhielt. 1953 wurde mit der BCG-Impfung gegen Tuberkulose eine weitere Impfung zur Pflicht, 1961 folgten die Schutzimpfungen gegen Poliomyelitis, ferner die Zweifachimpfung gegen Diphtherie und Tetanus, ab 1964 ergänzt um Pertussis als Dreifachimpfung. 1970 erhielt als letzte Impfung jene gegen Masern den Pflichtstatus [10]. Der Katalog der Pflichtimpfungen war damit komplettiert. Für den Einbezug weiterer Impfungen, etwa gegen Mumps oder Röteln, fehlten die zur Akquise der Impfseren notwendigen finanziellen Mittel. Da Letzteres auch die Beschaffung von Kombinationsimpfstoffen einschränkte, blieb die Zahl der wahrzunehmenden Impftermine stets hoch.

Seit 1955 war zur Aufnahme in staatliche Kindergärten und Krippen ein Impfnachweis gegen Pocken, Keuchhusten, Diphtherie, Tetanus und Tuberkulose vorzulegen, was einer impliziten Impfverpflichtung gleichkam. Ferner mussten Eltern prospektiv einer Verabreichung von Pflichtimpfungen im Rahmen des Besuchs der Einrichtungen zustimmen [13].

Grundlage für die Festlegung offizieller Pflichtimpfungen war zunächst die „Anordnung zur Durchführung von Schutzimpfungen“ vom 01.06.1949, die Verwaltungsorgane ermächtigte, Pflichtimpfungen anzuordnen. 1965 folgte das „Gesetz zur Verhütung und Bekämpfung übertragbarer Krankheiten“ [13]. Damit war die Impfpflicht zwar gesetzlich legitimiert, in der Praxis scheint es jedoch erhebliche Handlungsspielräume gegeben zu haben. Ausnahmen und Zurückstellungen waren verbreitet; Geldstrafen wurden nur selten tatsächlich verhängt [1]. Die Wahrnehmung eines Impftermins stellte eine Rechtspflicht dar, doch war bei Nichterscheinen „vor Erstattung einer Strafanzeige in jedem Falle eine mündliche Aussprache mit dem Impfverweigerer“ zu suchen, mahnte die SHI bereits 1958 [14]. Zwar gestand § 44 des Gesetzes der zuständigen Hygieneinspektion die zwangsweise Durchsetzung von Pflichtimpfungen zu, doch sah die Rechtsliteratur hierfür außerhalb unmittelbarer Seuchengefahr keinen Raum [15]. Belege für Zwangsvorführungen im Zusammenhang mit Pflichtimpfungen ließen sich bisher nicht finden. Das 1982 neugefasste „Gesetz zur Verhütung und Bekämpfung übertragbarer Krankheiten“ hätte dafür keine Grundlage mehr geboten [15]. Die Ablehnung einer Impfung wurde als Revers protokolliert und im MfG hinterlegt. Mehr konnte von den anhaltend überlasteten Bezirks- und Kreishygienikern, bei denen die Nachverfolgungspflicht lag, offensichtlich auch nicht erwartet werden. Konsequente Verfolgung von Impfverweigerung hätte pro Fall ausufernde Dokumentation, Strafanzeigenstellung bei der Volkspolizei bis hin zur Einleitung juristischer Maßnahmen bedeutet [1]. Dass ungeachtet dessen implizite bzw. subtilere Maßnahmen zur Durchsetzung der Impfpflicht angewandt wurden, zeigt der oben erwähnte Impfnachweis bei der Aufnahme in Kinderbetreuungseinrichtungen. Überdies hatte die SED-Führung 1952 die Verwaltungsgerichte in der DDR abgeschafft, vor denen Bürger staatliche Maßnahmen oder Vorgehensweisen rechtlich hätten überprüfen lassen können.

Abgesehen vom Problem der Impfmüdigkeit der Bevölkerung kannte auch das ärztliche Engagement auf diesem Feld Grenzen. Für die Vornahme von Impfungen war eine staatliche Befähigung notwendig. Diese wurde im Rahmen eines Lehrgangs der zuständigen Hygieneinspektion erworben und war alle drei Jahre zu erneuern [13]. Wer die Impfbefähigung erlangt hatte, sah sich einem großen Aufgabenbereich gegenüber. Aus Vorbeugemaßnahmen wie „Poliomyelitis-Schutzimpfung, BCG-Schutzimpfung, Schwangeren-Fürsorge, Mütterberatung und dergleichen“ entstand eine „erhebliche Mehrarbeit“, so ein Bezirksarzt [16]. Eine bessere Vergütung war damit nicht verbunden. Verstärkt wurde die Arbeitslast durch die Abwanderung ärztlichen Personals gen Westdeutschland. In ihrer Not beantragten Gesundheitseinrichtungen und ganze Kreise der DDR die Ausweitung der Arbeitszeiten des angestellten Gesundheitspersonals über 75 Wochenstunden hinaus, um die Erfüllung staatlicher Ziele und die gesundheitliche Versorgung der Bevölkerung zu garantieren [17].

Sorgen vor juristischen Konsequenzen bei Impfschädigungen trugen ebenfalls dazu bei, dass Medizinerinnen und Mediziner sich gegen die Impftätigkeit entschieden [1]. Bereits mit der Anordnung von 1949 wurde die Haftung der damaligen Länder „ohne Rücksicht auf Verschulden für Impfschädigungen“ festgelegt. Diese liberale Formulierung ermöglichte es Betroffenen, auch ohne abschließende Klärung der Schuldfrage Entschädigung zu erhalten. Anfang der 1950er-Jahre wurde der Umgang mit Impfschäden präzisiert, der patientenfreundliche Ansatz aber im Grundsatz beibehalten. Bei Nachweis von impfärztlichen Pflichtverletzungen oder mangelnder staatlicher Kontrolle des Impfserums haftete der Bezirk für entstandene Schäden. Lag ärztliches Verschulden vor, so stand dem Bezirk gegen „die schuldige Person ein Rückgriffsrecht“ zu [18]. Prinzipiell waren Impfärzte bei Verabreichung staatlich angeordneter Impfungen über eine Globalversicherung der Deutschen Versicherungs-Anstalt Berlin abgesichert [19]. Mögliche Entschädigungen umfassten die Erstattung ärztlicher Behandlungskosten und Erwerbsausfälle bis hin zu etwaigen Umschulungskosten bei ansonsten drohender Berufsunfähigkeit. In Härtefällen konnte die Ersatzleistung bis zu 10.000 DM betragen. Die Höhe entschied zunächst ein auf Kreisebene von der Abteilung Gesundheits- und Sozialwesen beauftragter Spruchausschuss [18]. Ab Mitte der 1960er-Jahre oblag diese Beurteilung Kommissionen, die auf Bezirksebene gebildet wurden [20]. Die finanziellen Entschädigungsmittel stellte das MfG [18].

## Der teure Kampf gegen die Poliomyelitis

Im Spätsommer 1953 brach in Leipzig eine größere Poliomyelitis-Epidemie aus. Binnen kurzer Zeit erkrankten 2663 Menschen, 141 von ihnen starben [21]. Die Ausmaße des Krankheitsausbruches und eine innenpolitisch fragile Lage nach dem erst wenige Wochen zurückliegenden Volksaufstand vom 17.06. ließen die Leipziger Epidemie zum Ausgangspunkt organisierter Bekämpfungsmaßnahmen seitens der DDR-Staatsführung werden. Die fäkal-oral übertragbare Viruserkrankung mit ihrem hohen Prozentsatz an klinisch stummen Ausscheidern beschäftigte in den 1950er-Jahren nicht nur das ostdeutsche Gesundheitswesen, sondern viele Länder dies- und jenseits des Eisernen Vorhangs [22, 23]. Besonders gefürchtet waren die schlaffen Paralysen, die der Kinderlähmung ihren Namen gaben und bei Befall der Atemmuskulatur lebensbedrohend waren. Die Therapie, die bis zur Langzeitbeatmung in Eisernen Lungen reichen konnte, war lediglich symptomatisch, dabei aber teuer und ressourcenintensiv.

Umso größer waren die Hoffnungen, die sich auf den 1955 in den USA zugelassenen Salk-Impfstoff^2^ richteten. Nachdem sich die Bundesrepublik 1957 zur Einführung des Totimpfstoffs entschlossen hatte, entschied man sich auch in Ost-Berlin für das Vakzin und organisierte für 1958 eine freiwillige Impfung der Geburtsjahrgänge 1954 bis 1956 [24]. Da die kurzfristige Beschaffung der benötigten Impfstoffchargen aus sozialistischen Staaten scheiterte, fiel die Wahl auf einen Impfstoff des Schweizer Serum- und Impfstoffinstitutes in Bern [25]. Die hohen Importkosten führten dazu, dass bereits die Planungsphase der Impfaktion von ökonomischen Zwängen geprägt war. „Entscheidend für die Frage, wieviel Personen insgesamt geimpft werden sollen, dürfte wahrscheinlich die Geldfrage … sein“, resümierte 1957 der Internist und beratende Sachverständige des MfG in Poliofragen, Albert Kukowka [24].

**Abb. 1:**
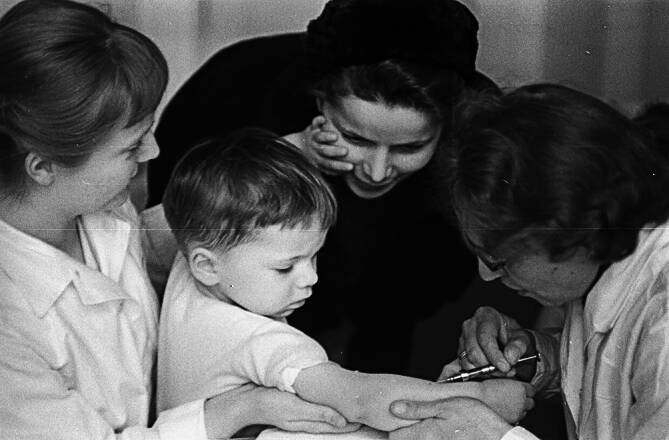
Ein zweijähriges Kind wird von einer Ärztin mit dem Salk-Impfstoff gegen Poliomyelitis geimpft, Gesundheitsamt Berlin-Pankow, 24.03.1958 (Bundesarchiv, Zentralbild Ulmer)

Um möglichst impfstoffsparend zu arbeiten, sollte die Injektion intrakutan statt intramuskulär verabreicht werden [22]. Diese nach ihrem Entwicklungsland auch als „Dänische Methode“ bezeichnete Applikationsform war umstritten, da man aufgrund der Verringerung der zugeführten Antigenmenge eine herabgesetzte Schutzwirkung befürchtete. Zudem war trotz impfstoffsparender Applikation absehbar, dass nur für 600.000 von 809.000 Kindern der Jahrgänge 1954 bis 1956 Impfstoff vorhanden sein würde [26]. Nicht zuletzt fehlte den meisten impfenden Ärztinnen und Ärzten die Erfahrung mit der intrakutanen Impftechnik, die eine sorgfältige und präzise Injektion in die obersten Hautschichten erfordert, um ausreichende Antikörperbildung zu provozieren [27]; Abb. 1 dokumentiert die intrakutane Polioimpfung.

Die Lösung des Problems ging vom „Fachausschuss für Poliomyelitis“ aus, einem Sachverständigengremium, das seit 1954 das MfG beriet. Der Ausschuss empfahl, dass nicht in erster Linie Ärztinnen und Ärzte, sondern Tuberkulosefürsorgerinnen die aufwendige Injektionsmethode anwenden sollten. Diese waren im Vergleich zum ärztlichen Personal in größerer Zahl verfügbar und mit der intrakutanen Methode insofern vertraut, als sie sie bei Tuberkulin-Hauttestungen im Rahmen von Reihenuntersuchungen anwandten. Die Tuberkulosefürsorgerinnen durften zudem seit 1953 bei ausgeprägtem örtlichen Personalmangel nach ärztlicher Delegierung auch die BCG-Impfungen vornehmen [28]. Damit war eine pragmatische Lösung gefunden, zugleich aber das ärztliche Primat beim Impfen an einer weiteren Stelle aufgegeben worden. Anders als bei heutigen Diskussionen über die Zulässigkeit der Delegation ärztlicher Tätigkeiten gab es in der DDR keine ärztlichen Standesvertretungen, die sich gegen den Verlust ärztlicher Vorrechte hätten stemmen können.

Nicht selten mangelte es an qualitativ hochwertigen Impfutensilien. Undichte Spritzenkolben und rissige Lötstellen an den eigens für die intrakutane Injektion verstärkten Kanülen führten zu Verlusten bei der ohnehin knappen Salk-Vakzine [29]. Immerhin konnte diese ab dem Frühjahr 1959 aus der Sowjetunion und damit kostengünstiger importiert werden. Die geringeren Beschaffungskosten ermöglichten eine Ausweitung und verstärkte Propagierung der Impfaktion [30]. Nach Meldungen von Durchbruchsinfektionen bei geimpften Kindern entschloss man sich zur Anpassung des Impfschemas: Als Kompromiss zwischen Schutzwirkung und Impfstoffverbrauch wurden nun intrakutane mit intramuskulären Injektionen kombiniert [31]. Ab Herbst 1959 wurde ausschließlich intramuskulär und damit nur noch von ärztlicher Hand geimpft [32].

Die Bilanz der Schutzimpfung nach Salk fiel verhalten aus. Das Ziel, mehr als die Hälfte der aufgerufenen Jahrgänge dreimal und somit vollständig zu immunisieren, war nicht erreicht worden. Als Hauptgründe für die niedrige Impfbeteiligung in der DDR wurden die Freiwilligkeit der Impfung sowie der Applikationsmodus per Spritze genannt [33]. Unerwähnt blieb, dass auch Material- und Personalmängel eine weitergehende Durchimpfung der Zielgruppe erschwert hatten, trotz Lockerung des impfärztlichen Primats. Letztlich trat die erhoffte epidemiologische Wirkung der Salk-Impfung nicht ein. Dies war jedoch nicht nur in der DDR der Fall. Auch in der Bundesrepublik und anderswo blieben die Ergebnisse der Salk-Impfung hinter den Erwartungen zurück. Neben der Impfzurückhaltung der Bevölkerung sah man dafür auch den Wirkmechanismus der Vakzine mittels inaktivierter Polioviren als ursächlich an [34]. Erst mit der Einführung der oralen Lebendvakzine nach Sabin-Tschumakow im Jahr 1960 konnte die Poliomyelitis in der DDR und andernorts effektiv bekämpft werden.

Die in den USA entwickelte und in der Sowjetunion umfassend erprobte orale Poliovakzine (OPV) zeichnete sich durch einfache Anwendung und geringe Herstellungskosten aus [23]. Anhaltender Diskussionsgegenstand bei Anwendung der OPV war ihre Sicherheit. Durch enterale Ausscheidung der Impfviren ergaben sich im Umfeld der Impflinge akzidentelle Viruskontakte. Während einige dies pragmatisch als indirekte Mitimpfung der Kontaktpersonen und weiteren Beitrag zum Herdenschutz betrachteten, fürchteten andere aufgrund der rezidivierenden Darmpassagen und der anhaltenden Vermehrung eine gesteigerte Virulenz der Polioviren [35]. Um die Zirkulation der Impfviren in der Bevölkerung möglichst kurz zu halten und Virulenzsteigerungen vorzubeugen, entwickelte Tschumakow bei seinen Anwendungsversuchen in der Sowjetunion spezielle Impfempfehlungen. Impfaktionen mit OPV sollten konzertiert stattfinden und möglichst die gesamte poliovulnerable Bevölkerung umfassen.

**Abb. 2:**
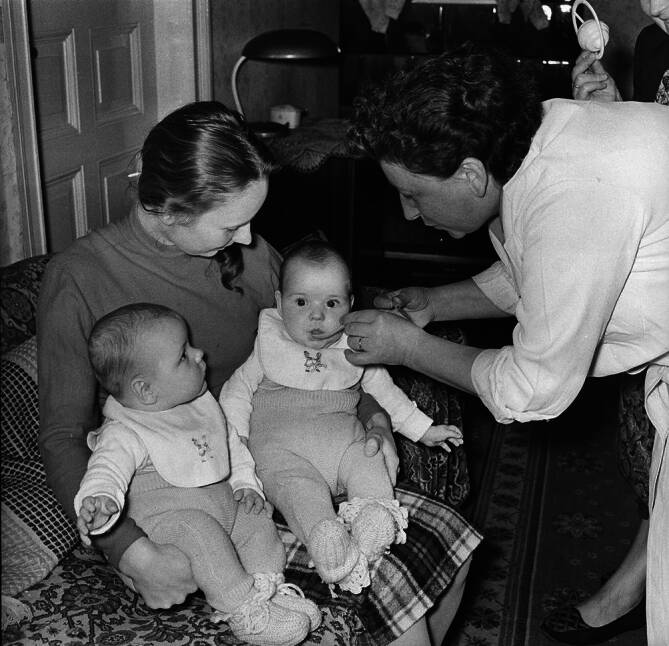
Aufsuchende Impfung: Eine Fürsorgerin vom Stadtbezirk Berlin-Weißensee verabreicht die Polio-Schluckimpfung in häuslicher Umgebung, 04.05.1960 (Bundesarchiv, Zentralbild Hochneder)

Bei Einführung der OPV in der DDR im Frühjahr 1960 folgten die Verantwortlichen diesen Empfehlungen. Über jeweils drei Tage wurden flächendeckend die besonders anfälligen Personen im Alter von zwei Monaten bis 20 Jahren immunisiert. Um die Impflinge kollektiv zu erfassen, hatte man die Verabreichung der Schluckimpfung aus den Dauerimpfstellen herausgelöst. Die Tropfen wurden direkt in Betreuungs- und Bildungseinrichtungen für Kinder und Jugendliche, an Universitäten sowie in Betrieben verabreicht. Dem in der Sowjetunion erprobten Prinzip der aufsuchenden Impfung folgend hatte man überdies „Impftrupps“ gebildet, die Menschen, die von den genannten Impfangeboten nicht erfasst wurden, direkt in deren Wohnungen aufsuchten und dort impften [3]. Um diese personalintensive Kampagne realisieren zu können, musste erneut vom impfärztlichen Primat abgewichen werden. Hatte man 1958 die Tuberkulosefürsorgerinnen herangezogen, so waren es nun Auszubildende der Gesundheitsberufe sowie vom Deutschen Roten Kreuz ausgebildete Gesundheitshelferinnen, denen die Durchführung der Schluckimpfung anvertraut wurde [36]; Abb. 2 dokumentiert eine aufsuchende Impfung, durchgeführt von einer Fürsorgerin.

Mit dem Einsatz der OPV starb die Kinderlähmung selbst einen schnellen Tod. In der DDR galt sie bereits 1961 als überwunden. Die Poliomyelitiszentren, die vordem in Erwartung weiter steigender Patientenzahlen auf Bezirksebene geplant oder bereits errichtet worden waren, wurden in der Folge nicht weitergebaut bzw. konnten sich den bereits erkrankten Patientinnen und Patienten widmen. Die damals oft neuartige Beatmungsausstattung nutzend gingen aus manchen Zentren anästhesiologische bzw. intensivmedizinische Abteilungen hervor, die es bis dato nicht gegeben hatte [37].

## Fazit

In den 1950er- und 1960er-Jahren entwarf die DDR Strategien, um die begrenzten Impfressourcen möglichst effizient einzusetzen. Zu den knappen Gütern zählten finanzielle Mittel, Impfutensilien und vor allem qualifiziertes Personal. Wir können zeigen, dass den impfenden Akteuren vor Ort ermöglicht wurde, bei Bedarf von normativen Vorgaben abzuweichen und pragmatische Lösungen zu suchen. Hierzu gehörten u. a. die Delegierung des Impfens an nichtärztliches Personal sowie die impfstoffsparende intrakutane Applikation. Zur Erzielung hoher Impfquoten nutzte das DDR-Gesundheitsministerium gezielt das Instrument von Leistungsvergleichen auf Kreis- und Bezirksebene. Zudem wurde das Konzept der aufsuchenden Impfung verfolgt. Ideologisch war das Impfen eng mit dem Erfolg des Sozialismus verknüpft; die Beteiligung an Impfkampagnen galt auch als Abbild der Zustimmung zum sozialistischen Gesellschaftsmodell. Gleichwohl gab es auch in der DDR-Bevölkerung Impfmüdigkeit oder -skepsis. Das gesichtete Aktenmaterial liefert keine Anhaltspunkte für eine scharfe Durchsetzung der Impfpflicht mit juristischen Mitteln oder gar für Zwangsimpfungen. Der Zugriff auf die Impflinge war demnach nicht so unmittelbar oder autoritär, wie man angesichts des repressiven Staatswesens annehmen könnte. Zumeist wurden subtilere Möglichkeiten zur Durchsetzung der Impfpflicht genutzt, etwa der Ausschluss von Kinderbetreuungseinrichtungen oder Ferienreisen. Ob auch andere Druckmittel angewandt wurden, wäre von der medizinhistorischen Forschung noch zu prüfen.

## Footnotes

1 BCG-Impfung: Imfpung mit Bacillus Calmette-Guérin, Lebendimpfstoff gegen Tuberkulose.

2 Totimpfstoff gegen Poliomyelitis, benannt nach seinem Entwickler, dem US-amerikanischen Arzt und Immunologen Jonas Salk (1914–1995).
